# Crystal structure of 1,10-phenanthrolinium violurate violuric acid penta­hydrate

**DOI:** 10.1107/S205698902401065X

**Published:** 2024-11-14

**Authors:** Rüdiger W. Seidel, Tsonko M. Kolev

**Affiliations:** a Institut für Pharmazie, Martin-Luther-Universität Halle-Wittenberg, Wolfgang-Langenbeck-Str. 4, 06120 Halle (Saale), Germany; b Institute of Molecular Biology "Roumen Tsanev", Bulgarian Academy of Sciences, Acad. G. Bonchev-Str. Bl. 21, Sofia 1113, Bulgaria; Vienna University of Technology, Austria

**Keywords:** 1,10-phenanthroline, violuric acid, proton-transfer compound, hydrogen bonding, co-crystal, crystal structure

## Abstract

The title compound [systematic name: 1,10-phenanthrolinium 6-hy­droxy-5-(oxido­imino)-1,3-diazinane-2,4-dione–6-hy­droxy-5-(hy­droxy­imino)-1,3-diazin­ane-2,4-dione–water (1/1/5)], C_12_H_9_N_2_^+^·C_4_H_2_N_3_O_4_^−^·C_4_H_3_N_3_O_4_·5H_2_O, is a co-crystal salt hydrate comprising 1,10-phenanthrolinium cations, violurate anions, free violuric acid as co-former and five water mol­ecules of crystallization per formula unit. The violurate and the violuric acid residues each form distinct N—H⋯O hydrogen-bonded tapes with a common *R*^2^_2_(8) hydrogen-bond motif extending parallel to (103). Solvent water mol­ecules connect the tapes to form a tri-periodic hydrogen-bonded network with channels extending parallel to the *a*-axis direction, which accommodate the N—H⋯O_water_ hydrogen-bonded 1,10-phenanthrolinium cations. Direct N—H⋯O hydrogen bonds between the 1,10-phenanthrolinium and violurate ions are not encountered.

## Abstract 2

The crystal structure of the co-crystal salt solvate 1,10-phenanthrolinium violurate violuric acid penta­hydrate features a tri-periodic hydrogen-bonded network with the violurate and violuric acid residues each assembled into tapes and the phenanthrolinium cations residing in channels.

## 1. Chemical context

Violuric acid (systematic name: 6-hy­droxy-5-nitroso-1*H*-pyrimidine-2,4-dione) is a derivative of barbituric acid and was first described by the German chemist Adolf von Baeyer more than 150 years ago (Baeyer, 1863 ▸). While free violuric acid is colourless, violurate salts typically exhibit an intense colour (Liebing *et al.*, 2019 ▸, and references therein). Coloured organic salts of violuric acid were reported as early as in 1909 (Hantzsch & Issaias, 1909 ▸; Zerewitinoff, 1909 ▸), but their crystal structures have only been investigated since 2006 (for more details, see: Section 4).

For the system violuric acid, 1,10-phenanthroline as an organic base and water as solvent, a *pK_a_*_1_ value of 4.35 can be assumed for violuric acid (Moratal *et al.*, 1985 ▸) and a *pK_a_* value of 4.84 for the conjugate acid of 1,10-phenanthroline (Haynes, 2016 ▸). Hence, we can estimate Δ*pK_a_* = *pK_a_*(protonated base) – *pK_a_*(acid) = 4.84 – 4.35 = 0.49. In the Δ*pK_a_* range between −1 and 4, the position of the acid proton, and thus the formation of a salt or a co-crystal (Aitipamula *et al.*, 2012 ▸), is difficult to predict (Cruz-Cabeza, 2012 ▸). In fact, the title compound represents a multicomponent crystal that can be regarded as a co-crystal salt hydrate, C_12_H_9_N_2_^+^·C_4_H_2_N_3_O_4_^−^·C_4_H_3_N_3_O_4_·5H_2_O.
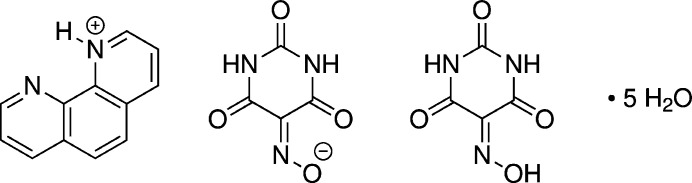


## 2. Structural commentary

**Figure 1:**
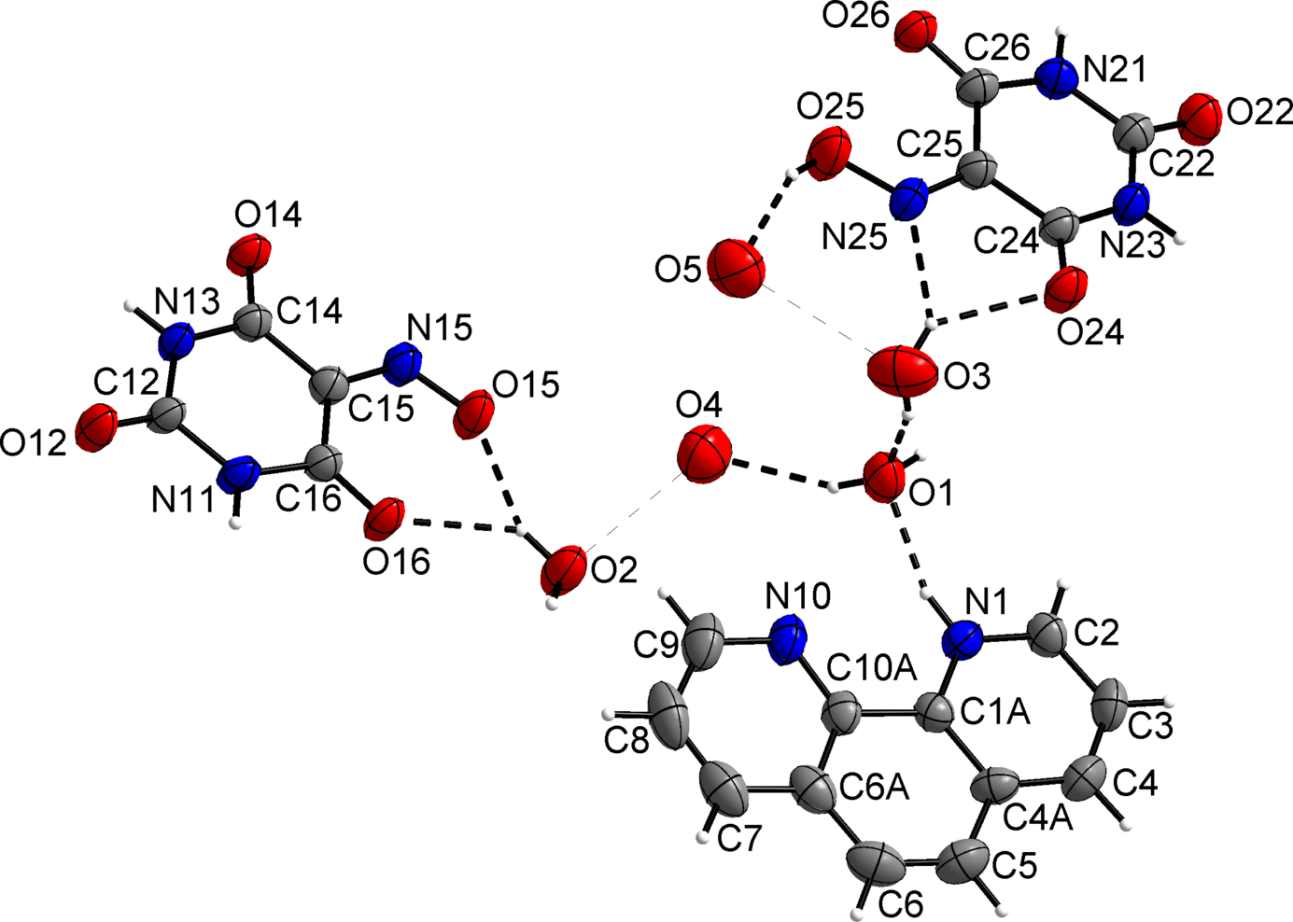
The asymmetric unit of the title compound, showing the mol­ecular entities with displacement ellipsoids drawn at the 50% probability level. Hydrogen atoms are shown as small spheres of arbitrary radius, and dashed lines represent hydrogen bonds. The water hydrogen atoms bound to O4 and O5 could not be located unambiguously and were therefore excluded from the structure model.

The asymmetric unit (Fig. 1 ▸) comprises a 1,10-phenanthrolinium cation, a violurate anion, a co-crystallized violuric acid mol­ecule and five water mol­ecules of crystallization (for two of the water mol­ecules, associated with O4 and O5, hydrogen atoms could not be located). Thus, the title compound represents a multicomponent crystal with eight independent residues (*Z*^R^ = 8; Grothe *et al.*, 2016 ▸). The parameter *Z*^R^, *i.e.* the number of crystallographically independent mol­ecules of any type is also known as *Z′′* (Steed & Steed, 2015 ▸). Inspired by the work by Aitipamula *et al.* (2012 ▸), Grothe *et al.* (2016 ▸) proposed a classification system for multicomponent crystals comprising seven categories. Accordingly, the title compound belongs to the class *co-crystal salt solvates*, which necessarily exhibit *Z*^R^ ≥ 4.

In the phenanthrolinium cation, the C2—N1—C1*A* angle is significantly larger by 6.4° than the C9—N10—C10*A* angle (Table 1 ▸), which corroborates the assignment of the site of protonation at N1. Likewise, the N—O and C—N bond lengths in the oxime (C25=N25—O25—H25) and the oximate (C15=N15—O15) moieties of the violuric acid and the violurate residue (Fig. 1 ▸; Table 1 ▸), lend support to the assignments of the sites of protonation and deprotonation, respectively.

## 3. Supra­molecular features

**Figure 2:**
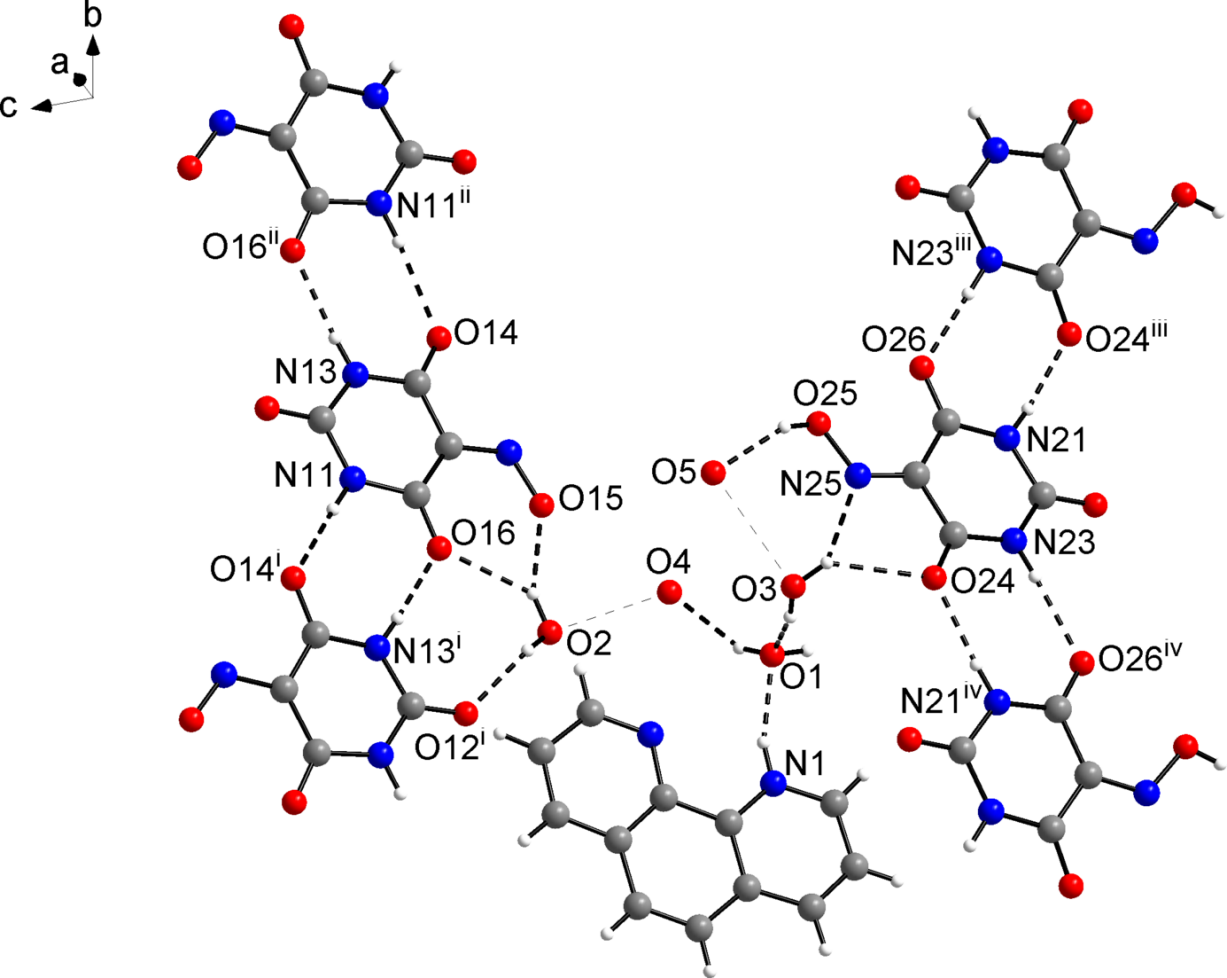
Section of the crystal structure of the title compound (viewed approximately along the *a-*axis direction towards the origin), illustrating some of the key hydrogen-bonding features (dashed lines); symmetry codes refer to Table 2 ▸. The water hydrogen atoms bound to O4 and O5 could not be located unambiguously and were therefore excluded from the structure model.

The predominant supra­molecular features of the crystal structure are N—H⋯O and O—H⋯O hydrogen bonds (Fig. 2 ▸). Table 2 ▸ lists the corresponding hydrogen-bond parameters, which are within expected ranges (Thakuria *et al.*, 2017 ▸). The violurate and the violuric acid residues each form linear polymeric strands through N—H⋯O hydrogen bonds with a common 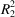
(8) motif (Allen *et al.*, 1999 ▸; Deepa *et al.*, 2014 ▸), extending parallel to the *b-*axis direction by application of the 2_1_ screw axis symmetry. Thus, there are two distinct hydrogen-bonded tapes, one of which features inter-anionic hydrogen bonds (Martín-Fernández *et al.*, 2024 ▸). Neutral and anionic hydrogen-bonded tapes stack in an alternating fashion parallel to the *a-*axis direction, with the mol­ecular planes extending parallel to (10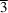).

These stacks of hydrogen-bonded tapes are separated by **c**/2 at *x*, *y*, 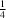 and *x*, *y*, 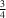, and are joined by the water mol­ecules through hydrogen-bonding, which results in an intricate tri-periodic network. The water mol­ecules are clustered at *x*, 0, 0 and *x*, 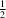, 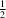 The water mol­ecule associated with O2 joins two violurate anions by a donating bifurcated hydrogen bond to the oximate oxygen atom O15 and the carbonyl oxygen O16, and a single O—H⋯O hydrogen bond to the carbonyl oxygen O12 of an adjacent mol­ecule. The water mol­ecule associated with O3 forms a donating bifurcated hydrogen bond to the carbonyl oxygen O24 and the oxime nitro­gen N25 of the neutral violuric acid mol­ecule, while the oxime hy­droxy group (O25) donates a hydrogen bond to the water oxygen atom O5. The donor functions of the latter and of O4 are unclear because their H atoms were not localized. However, the distances to possible acceptor O atoms indicate that there are several possibilities for hydrogen bonds of medium strength (Table 2 ▸).

**Figure 3:**
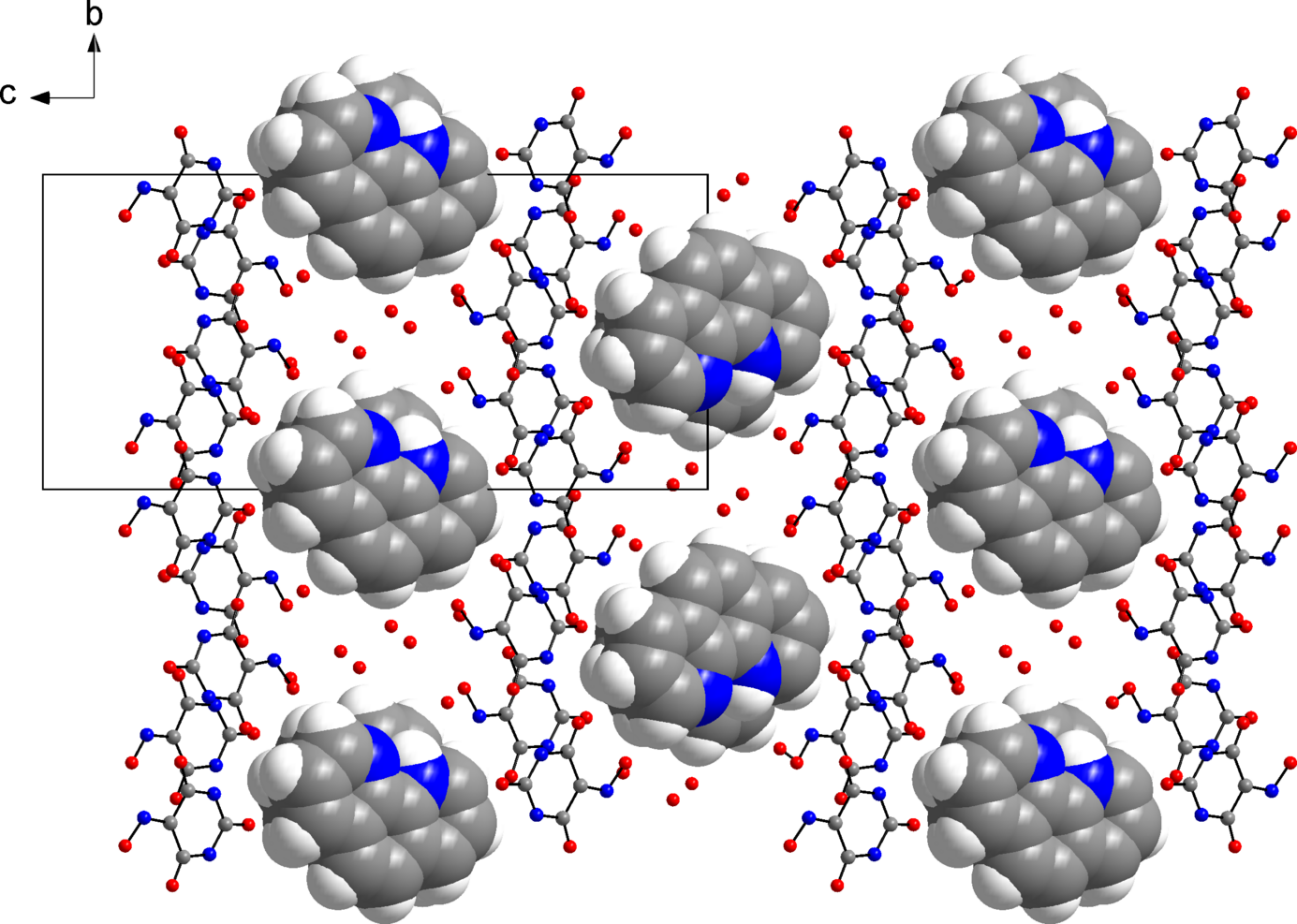
Packing diagram of the title compound viewed along the *a-*axis direction, showing the channel structure formed by the hydrogen-bonded network. Except for the phenanthrolinium cations (space-filling representation), hydrogen atoms were omitted for clarity. Colour scheme: C, grey; H, white; N, blue; O, red.

**Figure 4:**
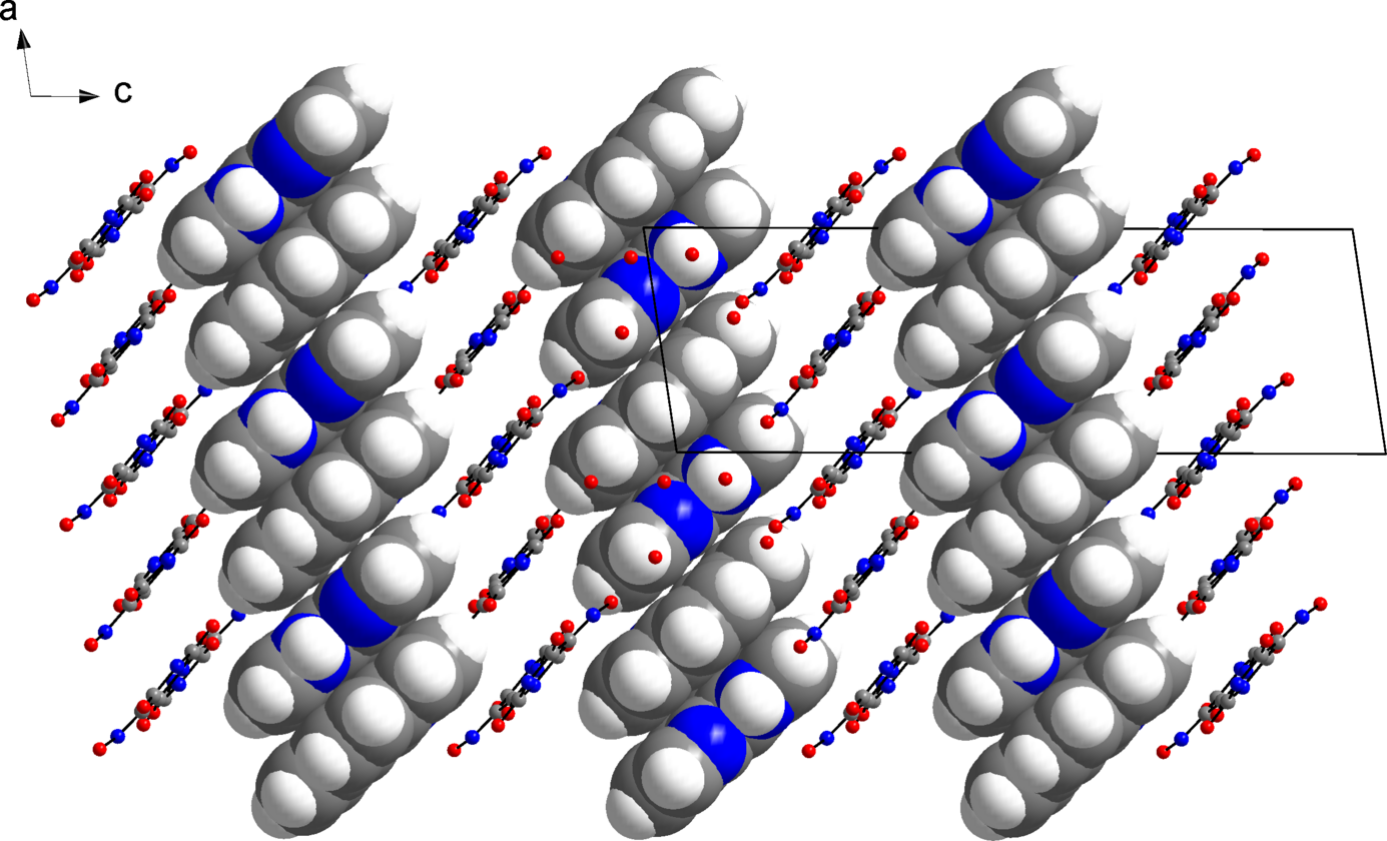
Packing diagram of the title compound viewed along the *b*-axis direction, showing the layer structure of phenanthrolinium cations, violurate anions and violuric acid mol­ecules. Representations and colour codes are as in Fig. 3 ▸.

Within the hydrogen-bonded network, the phenanthrolinium cations reside face-to-face stacked in channels extending parallel to the *a-*axis direction (Fig. 3 ▸), and each forms an N—H⋯O hydrogen bond to a water mol­ecule but neither to the violurate nor to violuric acid moieties. A view along the *b*-axis direction reveals a layered arrangement of the phenanthrolinium cations, violurate anions and violuric acid mol­ecules (Fig. 4 ▸). Within a stack, the mean planes through the phenanthrolinium ions related by inversion symmetry are separated by 3.46 and 3.55 Å in an alternating fashion.

## 4. Database survey

A search of the Cambridge Structural Database (CSD, version 5.45 with March 2024 updates; Groom *et al.*, 2016 ▸) revealed more than 60 entries for violuric acid or its monoanion (excluding metal-containing structures), of which some are duplicates. For the polymorphs of violuric acid monohydrate, see: Nichol & Clegg (2005*a* ▸) and Guille *et al.* (2007 ▸), and references cited therein. The structure of violuric acid methanol solvate was also reported by Nichol & Clegg (2005*b* ▸). The crystal structure of unsolvated free violuric acid is hitherto unknown, as far as we are able to ascertain. For the structure of ammonium violurate, see: Nichol & Clegg (2007 ▸), and for structures of multicomponent crystals of violuric acid and organic nitro­gen bases, see: Nichol & Clegg (2006 ▸), Kolev *et al.* (2009 ▸), Ivanova & Spiteller (2010 ▸), Ivanova *et al.* (2010 ▸), Koleva *et al.* (2010 ▸), Ivanova & Spiteller (2014 ▸), Liebing *et al.* (2019 ▸) and Ivanova & Spiteller (2019 ▸).

The structures most related to the title compound are piperidinium violurate sesquihydrate (CSD refcode: FUFPIG; Kolev *et al.*, 2009 ▸), 1,2,3,4-tetra­hydro­isoquinolinium violurate monohydrate (FUFPOM; Kolev *et al.*, 2009 ▸) and ephedrinium violurate dihydrate (WURCUI; Ivanova *et al.*, 2010 ▸), which likewise feature hydrogen-bonded tapes of violurate residues with an 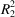
(8) motif, propagating by 2_1_ screw symmetry.

We note that the CSD also contains a variety of structures of violurate metal complexes, including alkali metal and alkaline earth metal salts. These are beyond the scope of this survey, and we direct the inter­ested reader to the review by Lorenz *et al.* (2019 ▸) for the coordination chemistry of violurate anions.

## 5. Synthesis and crystallization

1,10-Phenanthroline (170 mg, 0.94 mmol) and violuric acid (175 mg, 1.11 mmol) were mixed in 20 ml of water under continuous stirring at elevated temperature (323–353 K) for 24 h. A red precipitate was obtained after leaving the resulting solution at 298 K for about two weeks. The product was filtered off and air-dried. Red crystals of the title compound suitable for X-ray diffraction analysis were grown from a solution of the sample in doubly distilled water at room temperature over a period of three weeks.

## 6. Refinement

Crystal data, data collection and structure refinement details are summarized in Table 3 ▸. Carbon-bound H atoms and the oxime hy­droxy H25 atom were placed in geometrically calculated positions with *d*(C—H) = 0.93 Å and *d*(O—H) = 0.82 Å, respectively, and refined with a riding model. Nitro­gen-bound H atoms were located in a difference-Fourier map and their positions refined with the N—H distances restrained to a target value of 0.86 (2) Å. The water H atoms bound to O1, O2 and O3 were located in difference-Fourier maps, and the corresponding O—H distances were restrained to a target value of 0.82 (2) Å. The 1,3-H,H distances of the water mol­ecules were restrained to be similar with a standard deviation of 0.04 Å. *U*_iso_(H) was set 1.2*U*_eq_(C,N,O) for all H atoms. The water H atoms bound to O4 and O5 could not be located with certainty and were therefore excluded from the structural model, but are included in the chemical formula for calculation of crystal data. Two reflections (011 and 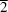
06) were obstructed by the beam stop and were omitted from the refinement.

## Supplementary Material

Crystal structure: contains datablock(s) I, global. DOI: 10.1107/S205698902401065X/wm5737sup1.cif

Structure factors: contains datablock(s) I. DOI: 10.1107/S205698902401065X/wm5737Isup2.hkl

Supporting information file. DOI: 10.1107/S205698902401065X/wm5737Isup3.cdx

Supporting information file. DOI: 10.1107/S205698902401065X/wm5737Isup4.cml

CCDC reference: 2396663

Additional supporting information:  crystallographic information; 3D view; checkCIF report

## Figures and Tables

**Table 1:** Selected geometric parameters (Å, °)

C15—N15	1.342 (5)	C25—N25	1.284 (5)
N15—O15	1.274 (4)	N25—O25	1.345 (4)
			
C2—N1—C1*A*	122.8 (4)	C9—N10—C10*A*	116.4 (4)

**Table 2:** Hydrogen-bond geometry (Å, °)

*D*—H⋯*A*	*D*—H	H⋯*A*	*D*⋯*A*	*D*—H⋯*A*
N1—H1⋯O1	0.88 (2)	1.89 (2)	2.730 (5)	161 (4)
N11—H11⋯O14^i^	0.88 (2)	2.12 (2)	2.997 (4)	173 (4)
N13—H13⋯O16^ii^	0.85 (2)	1.98 (2)	2.835 (4)	177 (4)
N21—H21⋯O24^iii^	0.87 (2)	1.99 (2)	2.858 (4)	172 (4)
N23—H23⋯O26^iv^	0.88 (2)	2.03 (2)	2.900 (4)	173 (4)
O1—H1*A*⋯O4	0.80 (2)	2.05 (3)	2.809 (5)	157 (5)
O1—H1*B*⋯O15^v^	0.85 (2)	1.87 (2)	2.719 (4)	177 (5)
O2—H2*A*⋯O12^i^	0.84 (2)	2.02 (3)	2.825 (4)	160 (5)
O2—H2*B*⋯O15	0.86 (2)	2.08 (3)	2.811 (5)	143 (4)
O2—H2*B*⋯O16	0.86 (2)	2.15 (4)	2.832 (4)	136 (4)
O3—H3*A*⋯N25	0.84 (2)	2.32 (4)	3.007 (5)	139 (5)
O3—H3*A*⋯O24	0.84 (2)	2.33 (4)	3.006 (5)	137 (5)
O3—H3*B*⋯O1	0.81 (2)	2.04 (4)	2.784 (6)	153 (6)
O25—H25⋯O5	0.82	1.92	2.692 (5)	156
O4⋯O2			2.694 (5)	
O4⋯O5^vi^			2.816 (6)	
O4⋯O4^v^			2.844 (7)	
O5⋯O5^vi^			2.837 (8)	
O5⋯O3			2.850 (6)	

Symmetry codes: (i) 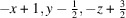; (ii) 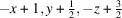; (iii) 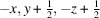; (iv) 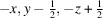; (v) 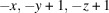; (vi) 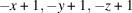.

**Table 3:** Experimental details

Crystal data	
Chemical formula	C_12_H_9_N_2_^+^·C_4_H_2_N_3_O_4_^−^·C_4_H_3_N_3_O_4_·5H_2_O
*M*_r_	584.47
Crystal system, space group	Monoclinic, *P*2_1_/*c*
Temperature (K)	294
*a*, *b*, *c* (Å)	8.247 (3), 12.0714 (16), 25.771 (4)
β (°)	98.572 (16)
*V* (Å^3^)	2537.1 (12)
*Z*	4
Radiation type	Mo *K*α
μ (mm^−1^)	0.13
Crystal size (mm)	0.29 × 0.25 × 0.22

Data collection	
Diffractometer	Siemens P4
No. of measured, independent and observed [*I* > 2σ(*I*)] reflections	5990, 4453, 2259
*R*_int_	0.050
(sin θ/λ)_max_ (Å^−1^)	0.595

Refinement	
*R*[*F*^2^ > 2σ(*F*^2^)], *wR*(*F*^2^), *S*	0.067, 0.187, 1.01
No. of reflections	4453
No. of parameters	404
No. of restraints	14
H-atom treatment	H atoms treated by a mixture of independent and constrained refinement
Δρ_max_, Δρ_min_ (e Å^−3^)	0.27, −0.21

Computer programs: *XSCANS* (Siemens, 1994 ▸), *SHELXS97* (Sheldrick, 2008 ▸), *SHELXL2019/3* (Sheldrick, 2015 ▸), *DIAMOND* (Brandenburg, 2018 ▸), *PLATON* (Spek, 2020 ▸), *enCIFer* (Allen *et al.*, 2004 ▸) and *publCIF* (Westrip, 2010 ▸).

## supplementary crystallographic information

### 1,10-Phenanthrolinium 6-hydroxy-5-(oxidoimino)-1,3-diazinane-2,4-dione–\ 6-hydroxy-5-(hydroxyimino)-1,3-diazinane-2,4-dione–water (1/1/5) Crystal data

**Table:**

C_12_H_9_N_2_^+^·C_4_H_2_N_3_O_4_^−^·C_4_H_3_N_3_O_4_·5H_2_O	*F*(000) = 1216
*M**_r_* = 584.47	*D*_x_ = 1.530 Mg m^−^^3^
Monoclinic, *P*2_1_/*c*	Mo *K*α radiation, λ = 0.71073 Å
*a* = 8.247 (3) Å	Cell parameters from 16 reflections
*b* = 12.0714 (16) Å	θ = 12.0–26.1°
*c* = 25.771 (4) Å	µ = 0.13 mm^−^^1^
β = 98.572 (16)°	*T* = 294 K
*V* = 2537.1 (12) Å^3^	Prism, red
*Z* = 4	0.29 × 0.25 × 0.22 mm

### 1,10-Phenanthrolinium 6-hydroxy-5-(oxidoimino)-1,3-diazinane-2,4-dione–\ 6-hydroxy-5-(hydroxyimino)-1,3-diazinane-2,4-dione–water (1/1/5) Data collection

**Table:**

Siemens P4 diffractometer	θ_max_ = 25.0°, θ_min_ = 2.3°
Radiation source: sealed X-ray tube	*h* = −9→1
ω scans	*k* = −1→14
5990 measured reflections	*l* = −30→30
4453 independent reflections	3 standard reflections every 15 min
2259 reflections with *I* > 2σ(*I*)	intensity decay: none
*R*_int_ = 0.050	

### 1,10-Phenanthrolinium 6-hydroxy-5-(oxidoimino)-1,3-diazinane-2,4-dione–\ 6-hydroxy-5-(hydroxyimino)-1,3-diazinane-2,4-dione–water (1/1/5) Refinement

**Table:**

Refinement on *F*^2^	Primary atom site location: structure-invariant direct methods
Least-squares matrix: full	Secondary atom site location: difference Fourier map
*R*[*F*^2^ > 2σ(*F*^2^)] = 0.067	Hydrogen site location: mixed
*wR*(*F*^2^) = 0.187	H atoms treated by a mixture of independent and constrained refinement
*S* = 1.01	*w* = 1/[σ^2^(*F*_o_^2^) + (0.0775*P*)^2^ + 0.7605*P*] where *P* = (*F*_o_^2^ + 2*F*_c_^2^)/3
4453 reflections	(Δ/σ)_max_ < 0.001
404 parameters	Δρ_max_ = 0.27 e Å^−^^3^
14 restraints	Δρ_min_ = −0.21 e Å^−^^3^

### 1,10-Phenanthrolinium 6-hydroxy-5-(oxidoimino)-1,3-diazinane-2,4-dione–\ 6-hydroxy-5-(hydroxyimino)-1,3-diazinane-2,4-dione–water (1/1/5) Special details

**Table:**

Geometry. All esds (except the esd in the dihedral angle between two l.s. planes) are estimated using the full covariance matrix. The cell esds are taken into account individually in the estimation of esds in distances, angles and torsion angles; correlations between esds in cell parameters are only used when they are defined by crystal symmetry. An approximate (isotropic) treatment of cell esds is used for estimating esds involving l.s. planes.

### 1,10-Phenanthrolinium 6-hydroxy-5-(oxidoimino)-1,3-diazinane-2,4-dione–\ 6-hydroxy-5-(hydroxyimino)-1,3-diazinane-2,4-dione–water (1/1/5) Fractional atomic coordinates and isotropic or equivalent isotropic displacement parameters (Å^2^)

**Table:**

	*x*	*y*	*z*	*U*_iso_*/*U*_eq_	
C1A	0.1694 (5)	−0.0134 (4)	0.46997 (15)	0.0351 (10)	
C2	−0.0174 (5)	0.0453 (4)	0.39698 (16)	0.0420 (11)	
H2	−0.067421	0.103132	0.376762	0.050*	
C3	−0.0579 (6)	−0.0633 (4)	0.38352 (18)	0.0492 (12)	
H3	−0.134885	−0.079078	0.354276	0.059*	
C4	0.0162 (6)	−0.1461 (4)	0.41353 (17)	0.0475 (12)	
H4	−0.009583	−0.219140	0.404186	0.057*	
C4A	0.1305 (5)	−0.1251 (3)	0.45819 (17)	0.0406 (11)	
C5	0.2093 (6)	−0.2071 (4)	0.49225 (19)	0.0565 (14)	
H5	0.185146	−0.281281	0.485187	0.068*	
C6	0.3184 (6)	−0.1803 (4)	0.5347 (2)	0.0582 (14)	
H6	0.367223	−0.236401	0.556320	0.070*	
C6A	0.3608 (5)	−0.0673 (4)	0.54709 (17)	0.0484 (12)	
C7	0.4758 (6)	−0.0371 (5)	0.58985 (18)	0.0604 (15)	
H7	0.530675	−0.090678	0.611739	0.072*	
C8	0.5062 (6)	0.0729 (6)	0.59896 (19)	0.0675 (16)	
H8	0.580971	0.095392	0.627666	0.081*	
C9	0.4234 (6)	0.1524 (5)	0.56446 (19)	0.0617 (15)	
H9	0.446266	0.226869	0.571235	0.074*	
C10A	0.2859 (5)	0.0173 (4)	0.51489 (17)	0.0403 (11)	
C12	0.5769 (5)	0.7219 (3)	0.78082 (16)	0.0367 (10)	
C14	0.3737 (5)	0.8194 (3)	0.71811 (15)	0.0349 (10)	
C15	0.3164 (5)	0.7134 (3)	0.69627 (15)	0.0356 (10)	
C16	0.3938 (5)	0.6117 (3)	0.71692 (16)	0.0360 (10)	
N11	0.5144 (4)	0.6237 (3)	0.76025 (14)	0.0404 (9)	
H11	0.560 (5)	0.561 (2)	0.7729 (15)	0.048*	
N13	0.5025 (5)	0.8154 (3)	0.75876 (14)	0.0394 (9)	
H13	0.546 (5)	0.875 (2)	0.7721 (15)	0.047*	
N15	0.1919 (4)	0.7205 (3)	0.65645 (13)	0.0432 (9)	
C22	−0.1051 (6)	0.5632 (4)	0.22527 (16)	0.0419 (11)	
C24	0.1034 (5)	0.4561 (3)	0.28192 (16)	0.0361 (10)	
C25	0.1608 (5)	0.5592 (3)	0.30845 (16)	0.0389 (11)	
C26	0.0898 (6)	0.6672 (3)	0.28899 (17)	0.0408 (11)	
N1	0.0931 (4)	0.0670 (3)	0.43884 (14)	0.0389 (9)	
H1	0.117 (5)	0.1369 (18)	0.4449 (16)	0.047*	
N10	0.3159 (5)	0.1267 (3)	0.52332 (14)	0.0494 (10)	
N21	−0.0407 (5)	0.6588 (3)	0.24939 (14)	0.0428 (10)	
H21	−0.088 (5)	0.719 (2)	0.2359 (15)	0.051*	
N23	−0.0273 (5)	0.4667 (3)	0.24254 (14)	0.0437 (10)	
H23	−0.054 (5)	0.403 (2)	0.2268 (15)	0.052*	
N25	0.2728 (5)	0.5426 (3)	0.34791 (14)	0.0478 (10)	
O1	0.1190 (4)	0.2924 (3)	0.43698 (12)	0.0570 (10)	
H1A	0.115 (6)	0.317 (4)	0.4657 (10)	0.068*	
H1B	0.039 (4)	0.314 (4)	0.4145 (14)	0.068*	
O2	0.1342 (5)	0.3996 (3)	0.62720 (15)	0.0669 (11)	
H2A	0.204 (5)	0.353 (3)	0.640 (2)	0.080*	
H2B	0.176 (6)	0.462 (2)	0.638 (2)	0.080*	
O3	0.4014 (5)	0.3233 (4)	0.39153 (19)	0.0802 (12)	
H3A	0.372 (7)	0.364 (4)	0.3655 (16)	0.096*	
H3B	0.324 (5)	0.293 (5)	0.402 (2)	0.096*	
O4	0.1337 (5)	0.4343 (3)	0.52391 (14)	0.0822 (12)	
O5	0.5317 (5)	0.5145 (3)	0.44781 (14)	0.0823 (12)	
O12	0.6901 (4)	0.7252 (2)	0.81696 (12)	0.0543 (9)	
O14	0.3169 (4)	0.9110 (2)	0.70432 (11)	0.0466 (8)	
O15	0.1345 (4)	0.6320 (2)	0.63379 (12)	0.0563 (9)	
O16	0.3588 (4)	0.5181 (2)	0.70065 (12)	0.0480 (8)	
O22	−0.2202 (4)	0.5645 (3)	0.19080 (12)	0.0596 (10)	
O24	0.1668 (4)	0.3658 (2)	0.29265 (12)	0.0484 (8)	
O25	0.3305 (5)	0.6316 (3)	0.37618 (14)	0.0714 (11)	
H25	0.407415	0.612960	0.398400	0.086*	
O26	0.1407 (4)	0.7577 (2)	0.30519 (12)	0.0496 (9)	

### 1,10-Phenanthrolinium 6-hydroxy-5-(oxidoimino)-1,3-diazinane-2,4-dione–\ 6-hydroxy-5-(hydroxyimino)-1,3-diazinane-2,4-dione–water (1/1/5) Atomic displacement parameters (Å^2^)

**Table:**

	*U*^11^	*U*^22^	*U*^33^	*U*^12^	*U*^13^	*U*^23^
C1A	0.031 (2)	0.038 (2)	0.036 (2)	0.001 (2)	0.0031 (19)	0.002 (2)
C2	0.039 (3)	0.044 (3)	0.040 (2)	0.003 (2)	−0.004 (2)	0.001 (2)
C3	0.046 (3)	0.056 (3)	0.043 (3)	−0.008 (2)	−0.004 (2)	−0.012 (2)
C4	0.051 (3)	0.036 (3)	0.055 (3)	−0.006 (2)	0.007 (2)	−0.008 (2)
C4A	0.036 (3)	0.032 (2)	0.053 (3)	0.002 (2)	0.006 (2)	0.007 (2)
C5	0.058 (3)	0.039 (3)	0.072 (4)	−0.001 (3)	0.010 (3)	0.001 (3)
C6	0.057 (3)	0.056 (3)	0.063 (3)	0.014 (3)	0.013 (3)	0.018 (3)
C6A	0.039 (3)	0.064 (3)	0.041 (3)	0.006 (3)	0.003 (2)	0.008 (2)
C7	0.050 (3)	0.086 (4)	0.043 (3)	0.008 (3)	0.000 (2)	0.008 (3)
C8	0.046 (3)	0.111 (5)	0.041 (3)	−0.002 (3)	−0.006 (2)	−0.012 (3)
C9	0.053 (3)	0.073 (4)	0.057 (3)	−0.017 (3)	0.001 (3)	−0.017 (3)
C10A	0.033 (2)	0.047 (3)	0.040 (2)	−0.005 (2)	0.002 (2)	−0.003 (2)
C12	0.036 (2)	0.026 (2)	0.044 (2)	−0.003 (2)	−0.005 (2)	0.002 (2)
C14	0.038 (2)	0.028 (2)	0.037 (2)	0.0017 (19)	0.001 (2)	0.0002 (19)
C15	0.035 (2)	0.032 (2)	0.038 (2)	0.002 (2)	0.001 (2)	0.0001 (19)
C16	0.038 (2)	0.030 (2)	0.038 (2)	−0.002 (2)	0.000 (2)	0.002 (2)
N11	0.041 (2)	0.025 (2)	0.050 (2)	−0.0004 (17)	−0.0092 (18)	0.0050 (17)
N13	0.044 (2)	0.0263 (19)	0.042 (2)	−0.0032 (17)	−0.0122 (18)	−0.0005 (16)
N15	0.046 (2)	0.034 (2)	0.044 (2)	−0.0012 (18)	−0.0115 (18)	−0.0002 (17)
C22	0.048 (3)	0.035 (3)	0.041 (3)	0.000 (2)	0.002 (2)	−0.001 (2)
C24	0.037 (2)	0.029 (2)	0.041 (2)	0.000 (2)	0.002 (2)	0.001 (2)
C25	0.042 (3)	0.031 (2)	0.041 (2)	−0.001 (2)	−0.002 (2)	0.002 (2)
C26	0.048 (3)	0.029 (2)	0.044 (3)	0.002 (2)	0.003 (2)	0.000 (2)
N1	0.038 (2)	0.033 (2)	0.045 (2)	−0.0025 (18)	0.0027 (18)	−0.0018 (18)
N10	0.046 (2)	0.053 (3)	0.046 (2)	−0.010 (2)	−0.003 (2)	−0.010 (2)
N21	0.048 (2)	0.033 (2)	0.044 (2)	0.0075 (18)	−0.0046 (19)	0.0037 (18)
N23	0.052 (2)	0.030 (2)	0.044 (2)	−0.0008 (19)	−0.0091 (19)	−0.0052 (17)
N25	0.056 (2)	0.037 (2)	0.047 (2)	−0.0108 (19)	−0.005 (2)	−0.0056 (18)
O1	0.064 (2)	0.051 (2)	0.048 (2)	0.0016 (18)	−0.0149 (19)	−0.0008 (18)
O2	0.076 (3)	0.039 (2)	0.074 (3)	−0.0058 (19)	−0.028 (2)	−0.0016 (19)
O3	0.067 (3)	0.072 (3)	0.098 (3)	0.015 (2)	0.002 (2)	0.032 (2)
O4	0.105 (3)	0.064 (2)	0.074 (3)	0.003 (2)	0.003 (2)	−0.008 (2)
O5	0.077 (3)	0.086 (3)	0.077 (3)	0.000 (2)	−0.014 (2)	0.014 (2)
O12	0.056 (2)	0.0371 (18)	0.059 (2)	−0.0008 (16)	−0.0269 (17)	0.0014 (15)
O14	0.055 (2)	0.0242 (16)	0.0548 (19)	0.0045 (15)	−0.0115 (16)	0.0014 (14)
O15	0.063 (2)	0.0365 (18)	0.058 (2)	−0.0032 (17)	−0.0255 (17)	−0.0053 (16)
O16	0.057 (2)	0.0224 (16)	0.0574 (19)	0.0007 (15)	−0.0162 (16)	−0.0039 (15)
O22	0.061 (2)	0.050 (2)	0.059 (2)	0.0040 (18)	−0.0194 (19)	−0.0003 (17)
O24	0.055 (2)	0.0269 (17)	0.058 (2)	0.0069 (15)	−0.0088 (16)	−0.0053 (15)
O25	0.084 (3)	0.047 (2)	0.069 (2)	−0.007 (2)	−0.033 (2)	−0.0034 (19)
O26	0.061 (2)	0.0300 (17)	0.0527 (19)	−0.0019 (16)	−0.0078 (16)	−0.0001 (15)

### 1,10-Phenanthrolinium 6-hydroxy-5-(oxidoimino)-1,3-diazinane-2,4-dione–\ 6-hydroxy-5-(hydroxyimino)-1,3-diazinane-2,4-dione–water (1/1/5) Geometric parameters (Å, º)

**Table:**

C1A—N1	1.353 (5)	C14—C15	1.449 (6)
C1A—C4A	1.408 (6)	C15—N15	1.342 (5)
C1A—C10A	1.438 (5)	C15—C16	1.448 (6)
C2—N1	1.330 (5)	C16—O16	1.224 (5)
C2—C3	1.384 (6)	C16—N11	1.388 (5)
C2—H2	0.9300	N11—H11	0.884 (19)
C3—C4	1.353 (6)	N13—H13	0.852 (19)
C3—H3	0.9300	N15—O15	1.274 (4)
C4—C4A	1.398 (6)	C22—O22	1.200 (5)
C4—H4	0.9300	C22—N23	1.372 (5)
C4A—C5	1.415 (6)	C22—N21	1.378 (5)
C5—C6	1.347 (7)	C24—O24	1.222 (5)
C5—H5	0.9300	C24—N23	1.372 (5)
C6—C6A	1.433 (7)	C24—C25	1.465 (6)
C6—H6	0.9300	C25—N25	1.284 (5)
C6A—C7	1.391 (6)	C25—C26	1.485 (6)
C6A—C10A	1.400 (6)	C26—O26	1.222 (5)
C7—C8	1.366 (7)	C26—N21	1.372 (5)
C7—H7	0.9300	N1—H1	0.876 (19)
C8—C9	1.413 (7)	N21—H21	0.873 (19)
C8—H8	0.9300	N23—H23	0.879 (19)
C9—N10	1.314 (6)	N25—O25	1.345 (4)
C9—H9	0.9300	O1—H1A	0.803 (19)
C10A—N10	1.355 (6)	O1—H1B	0.850 (19)
C12—O12	1.217 (4)	O2—H2A	0.837 (19)
C12—N13	1.367 (5)	O2—H2B	0.858 (19)
C12—N11	1.367 (5)	O3—H3A	0.841 (19)
C14—O14	1.232 (5)	O3—H3B	0.813 (19)
C14—N13	1.377 (5)	O25—H25	0.8200
			
N1—C1A—C4A	119.2 (4)	N13—C14—C15	115.7 (4)
N1—C1A—C10A	119.2 (4)	N15—C15—C16	125.5 (4)
C4A—C1A—C10A	121.6 (4)	N15—C15—C14	114.1 (4)
N1—C2—C3	120.0 (4)	C16—C15—C14	120.4 (3)
N1—C2—H2	120.0	O16—C16—N11	118.4 (4)
C3—C2—H2	120.0	O16—C16—C15	126.0 (4)
C4—C3—C2	119.0 (4)	N11—C16—C15	115.5 (4)
C4—C3—H3	120.5	C12—N11—C16	125.9 (3)
C2—C3—H3	120.5	C12—N11—H11	119 (3)
C3—C4—C4A	121.9 (4)	C16—N11—H11	115 (3)
C3—C4—H4	119.1	C12—N13—C14	126.4 (4)
C4A—C4—H4	119.1	C12—N13—H13	113 (3)
C4—C4A—C1A	117.1 (4)	C14—N13—H13	120 (3)
C4—C4A—C5	125.1 (4)	O15—N15—C15	119.1 (3)
C1A—C4A—C5	117.8 (4)	O22—C22—N23	122.1 (4)
C6—C5—C4A	121.7 (5)	O22—C22—N21	122.1 (4)
C6—C5—H5	119.2	N23—C22—N21	115.7 (4)
C4A—C5—H5	119.2	O24—C24—N23	120.9 (4)
C5—C6—C6A	121.4 (5)	O24—C24—C25	123.7 (4)
C5—C6—H6	119.3	N23—C24—C25	115.4 (4)
C6A—C6—H6	119.3	N25—C25—C24	112.4 (4)
C7—C6A—C10A	117.9 (5)	N25—C25—C26	127.3 (4)
C7—C6A—C6	122.8 (5)	C24—C25—C26	120.3 (4)
C10A—C6A—C6	119.4 (4)	O26—C26—N21	120.8 (4)
C8—C7—C6A	118.5 (5)	O26—C26—C25	124.9 (4)
C8—C7—H7	120.8	N21—C26—C25	114.4 (4)
C6A—C7—H7	120.8	C2—N1—C1A	122.8 (4)
C7—C8—C9	119.5 (5)	C2—N1—H1	116 (3)
C7—C8—H8	120.2	C1A—N1—H1	121 (3)
C9—C8—H8	120.2	C9—N10—C10A	116.4 (4)
N10—C9—C8	123.6 (5)	C26—N21—C22	127.2 (4)
N10—C9—H9	118.2	C26—N21—H21	119 (3)
C8—C9—H9	118.2	C22—N21—H21	113 (3)
N10—C10A—C6A	124.2 (4)	C22—N23—C24	126.7 (4)
N10—C10A—C1A	117.7 (4)	C22—N23—H23	121 (3)
C6A—C10A—C1A	118.2 (4)	C24—N23—H23	112 (3)
O12—C12—N13	122.5 (4)	C25—N25—O25	117.4 (4)
O12—C12—N11	121.8 (4)	H1A—O1—H1B	112 (4)
N13—C12—N11	115.7 (3)	H2A—O2—H2B	105 (4)
O14—C14—N13	117.9 (4)	H3A—O3—H3B	112 (5)
O14—C14—C15	126.3 (4)	N25—O25—H25	109.5
			
N1—C2—C3—C4	0.1 (7)	N13—C12—N11—C16	5.8 (6)
C2—C3—C4—C4A	1.0 (7)	O16—C16—N11—C12	174.6 (4)
C3—C4—C4A—C1A	−1.8 (7)	C15—C16—N11—C12	−7.6 (6)
C3—C4—C4A—C5	178.1 (5)	O12—C12—N13—C14	−179.7 (4)
N1—C1A—C4A—C4	1.5 (6)	N11—C12—N13—C14	−0.7 (7)
C10A—C1A—C4A—C4	−179.7 (4)	O14—C14—N13—C12	177.2 (4)
N1—C1A—C4A—C5	−178.4 (4)	C15—C14—N13—C12	−1.8 (6)
C10A—C1A—C4A—C5	0.3 (7)	C16—C15—N15—O15	−0.4 (7)
C4—C4A—C5—C6	180.0 (5)	C14—C15—N15—O15	178.7 (4)
C1A—C4A—C5—C6	−0.1 (7)	O24—C24—C25—N25	−7.2 (6)
C4A—C5—C6—C6A	−0.5 (8)	N23—C24—C25—N25	174.0 (4)
C5—C6—C6A—C7	−178.4 (5)	O24—C24—C25—C26	172.8 (4)
C5—C6—C6A—C10A	0.8 (7)	N23—C24—C25—C26	−6.0 (6)
C10A—C6A—C7—C8	1.7 (7)	N25—C25—C26—O26	7.6 (8)
C6—C6A—C7—C8	−179.0 (5)	C24—C25—C26—O26	−172.4 (4)
C6A—C7—C8—C9	−1.2 (8)	N25—C25—C26—N21	−173.4 (4)
C7—C8—C9—N10	0.3 (8)	C24—C25—C26—N21	6.6 (6)
C7—C6A—C10A—N10	−1.3 (7)	C3—C2—N1—C1A	−0.4 (7)
C6—C6A—C10A—N10	179.4 (5)	C4A—C1A—N1—C2	−0.4 (6)
C7—C6A—C10A—C1A	178.7 (4)	C10A—C1A—N1—C2	−179.2 (4)
C6—C6A—C10A—C1A	−0.6 (6)	C8—C9—N10—C10A	0.1 (7)
N1—C1A—C10A—N10	−1.2 (6)	C6A—C10A—N10—C9	0.4 (7)
C4A—C1A—C10A—N10	−179.9 (4)	C1A—C10A—N10—C9	−179.6 (4)
N1—C1A—C10A—C6A	178.8 (4)	O26—C26—N21—C22	176.0 (4)
C4A—C1A—C10A—C6A	0.0 (6)	C25—C26—N21—C22	−3.0 (7)
O14—C14—C15—N15	1.7 (7)	O22—C22—N21—C26	180.0 (5)
N13—C14—C15—N15	−179.4 (4)	N23—C22—N21—C26	−1.2 (7)
O14—C14—C15—C16	−179.1 (4)	O22—C22—N23—C24	−179.2 (4)
N13—C14—C15—C16	−0.3 (6)	N21—C22—N23—C24	2.0 (7)
N15—C15—C16—O16	1.1 (7)	O24—C24—N23—C22	−177.3 (4)
C14—C15—C16—O16	−177.9 (4)	C25—C24—N23—C22	1.6 (6)
N15—C15—C16—N11	−176.5 (4)	C24—C25—N25—O25	−177.9 (4)
C14—C15—C16—N11	4.5 (6)	C26—C25—N25—O25	2.1 (7)
O12—C12—N11—C16	−175.2 (4)		

### 1,10-Phenanthrolinium 6-hydroxy-5-(oxidoimino)-1,3-diazinane-2,4-dione–\ 6-hydroxy-5-(hydroxyimino)-1,3-diazinane-2,4-dione–water (1/1/5) Hydrogen-bond geometry (Å, º)

**Table:**

*D*—H···*A*	*D*—H	H···*A*	*D*···*A*	*D*—H···*A*
N1—H1···O1	0.88 (2)	1.89 (2)	2.730 (5)	161 (4)
N11—H11···O14^i^	0.88 (2)	2.12 (2)	2.997 (4)	173 (4)
N13—H13···O16^ii^	0.85 (2)	1.98 (2)	2.835 (4)	177 (4)
N21—H21···O24^iii^	0.87 (2)	1.99 (2)	2.858 (4)	172 (4)
N23—H23···O26^iv^	0.88 (2)	2.03 (2)	2.900 (4)	173 (4)
O1—H1*A*···O4	0.80 (2)	2.05 (3)	2.809 (5)	157 (5)
O1—H1*B*···O15^v^	0.85 (2)	1.87 (2)	2.719 (4)	177 (5)
O2—H2*A*···O12^i^	0.84 (2)	2.02 (3)	2.825 (4)	160 (5)
O2—H2*B*···O15	0.86 (2)	2.08 (3)	2.811 (5)	143 (4)
O2—H2*B*···O16	0.86 (2)	2.15 (4)	2.832 (4)	136 (4)
O3—H3*A*···N25	0.84 (2)	2.32 (4)	3.007 (5)	139 (5)
O3—H3*A*···O24	0.84 (2)	2.33 (4)	3.006 (5)	137 (5)
O3—H3*B*···O1	0.81 (2)	2.04 (4)	2.784 (6)	153 (6)
O25—H25···O5	0.82	1.92	2.692 (5)	156
O4···O2			2.694 (5)	
O4···O5^vi^			2.816 (6)	
O4···O4^v^			2.844 (7)	
O5···O5^vi^			2.837 (8)	
O5···O3			2.850 (6)	

Symmetry codes: (i) −*x*+1, *y*−1/2, −*z*+3/2; (ii) −*x*+1, *y*+1/2, −*z*+3/2; (iii) −*x*, *y*+1/2, −*z*+1/2; (iv) −*x*, *y*−1/2, −*z*+1/2; (v) −*x*, −*y*+1, −*z*+1; (vi) −*x*+1, −*y*+1, −*z*+1.
